# Elements of Pathological Anatomy

**Published:** 1857-09

**Authors:** 


ART. VII.—*Elements of Pathological Anatomy.* By SAMUEL D. GROSS, M. D., Prof. of Surgery in the Jefferson Medical College of Philadelphia, etc., etc. Third edition, modified and thoroughly revised. Illustrated by three hundred and forty-two Engravings on Wood. Philadelphia: BLANCHARD & LEA. 8vo., pp. 771. 1857.

The second edition of the treatise on the elements of pathological anatomy by Prof. Gross, appeared in 1845. During the twelve years that have elapsed, this field of scientific research, not less than other departments of medical knowledge, has undergone important changes due to its diligent, skillful cultivation. Continued study, especially by means of the microscope and chemico-anatomical investigations, has developed new facts and relations, increasing our knowledge both by freshly added truths and by expanding, confirming or correcting views which at that time seemed most consistent with the existing state of science. A new edition of the work, in the opinion of its author, was called for in order to render it a correct exponent of the elements of pathological anatomy at the present time; and, with Prof. Gross, to see before him a duty to be performed, is sufficient for him to enter at once upon its performance, without regard to the labor which it involves, and to apply himself unremittingly to the task till it is finished.

In the present edition, we have, in effect, a new treatise on the elements of pathological anatomy. Notwithstanding modifications and largely added matter, its size is somewhat less than that of the edition of 1845. The explanation is as follows: In the second edition much was introduced which was relevant to, and appropriately associated with the subjects embraced in pathological anatomy; but, yet, not intrinsically belonging to this department of science. Points relating to histology and diagnosis, occupied considerable space. This adjunctive matter has been omitted and the work restricted in its scope more rigidly within the boundaries of pathological anatomy. The extended plan of the second edition was more called for at the time of its publication than now, the points relinquished having, in the mean time, received more consideration than previously in other treatises. The space thus made vacant by excluded matter, left abundant room for the additions requisite in bringing the work up to the present state of science, at the same time, permitting a reduction in the size of the volume.

The task which the author imposed on himself in preparing a new edition of the work, was not a light one. It was widely different from that of sending forth a new edition with simply a few addenda. It involved a careful reconsideration of all the various subjects treated of, and a reconstruction of the treatise, the latter requiring much to be rewritten. None can adequately appreciate the labor of such an undertaking but they who have had some experience in bibliography. We congratulate the author on the completion of the task—a task devoid of the stimulus which we may suppose lightened the labor of writing the first edition, when reputation as an author was yet to be acquired. The desire of rendering a service to science and his profession must have been the chief source of encouragement during the many hours of drudging toil which the task required.

The general arrangement of the new, is the same as that of the preceding edition. The work is divided into two parts. Part *first*, embracing 192 pages, is devoted to the "general principles of pathological anatomy." In this division are considered, inflammation and the various processes incident thereto; transformations of structure; hypertrophy and atrophy, pneumatosis, polypes, hydatids, serous cysts, and the several heterologous formations. Part *second* is entitled "special pathological anatomy," and treats of the morbid changes occurring in the blood, the different tissues, anatomical systems, and the more important of the organs of the body.

Availing himself of the contributions which have been made of late years to pathological anatomy by Rokitansky, Hasse, Vogel and others; and also of the coöperation of Dr. Da Costa, of Philadelphia, whose labors in microscopical research already entitle him to high distinction, Prof. Gross has, in addition, embodied in this treatise much that is original. Formerly a public teacher of this branch of medicine, it has continued to occupy a portion of his studies since the publication of the first edition of the work in 1839. During this period he has collected and registered a large number of observations with which the pages of the present edition are enriched. The work, therefore, is by no means to be considered as merely a compilation. In this particular it may be contrasted favorably with a recent English treatise on the same department, of undoubted merit, but which is, in a great measure, an abridgement of the more elaborate work by Rokitansky.

The work by Prof. Gross is professedly elementary. The author does not aim to carry the reader to the farthest extent to which researches relating to the different subjects have been pushed. His object is to introduce the medical student to the pursuit of pathological anatomy, and to place before him those facts and considerations belonging to this province of science, in its present condition, which are practically most important. Such a work is, we think, needed. Pathological anatomy, which at one time was too exclusively cultivated, is at the present time, perhaps, in some danger of being unduly depreciated. True it is that morbid changes which our senses can appreciate, do not constitute the primary, essential abnormal aberrations of disease. There are perversions more occult which underlie the deviations from normal structure and the alterations in fluids which it is the province of the anatomist to study and describe. The appreciable anatomical characters of disease are the effects of ulterior, inappreciable disturbance of the conditions of health. True as this is, however, the sensible changes in the solids and fluids of the body are, and ever will be of fundamental importance as a basis not only of pathology but of practical medicine. It is, and always must be, a grand object for the medical and the surgical practitioner to recognize and discriminate these changes in the living organism; to know all that can be known, severally, respecting their nature, laws and tendencies, as the true point of departure for the application of remedial measures. Moreover, the facts developed by the study of morbid anatomy constitute the scaffolding by which the scientific inquirer may hope to ascend higher and higher toward the region in which rest the primary and essential truths of pathology. We concur fully in the author's remarks, in the preface, on the importance of the branch of which he treats, and on the insufficient degree of attention bestowed upon it in most of our medical schools. We trust that the publication of this treatise may lead to the result which he hopes for, viz., arousing the attention of the profession to the claims of pathological anatomy.

The numerous illustrations contained in this work enhance in no small degree its value. Of the three hundred and fifty-two wood engravings distributed in its pages, one hundred and thirty are newly added in the present edition. The larger portion of these are from drawings of specimens contained in the cabinet of the author and collected mainly from his own cases. This statement shows the extent to which he has contributed original matter in preparing the work.

In commending the work to the student and practitioner, we simply discharge a duty which the journalist owes, not alone to the author, but to the medical profession of this country. Duty alike to the profession and to the author, dictates something more than this. The bibliographical labors of Prof. Gross, reflecting the highest honor upon his talents, zeal and learning, involve a debt of gratitude which is not to be measured by the intrinsic merit of his publications, however great. A still deeper obligation attaches to the example which his career affords to the junior members of the medical profession. Beginning professional life without any exemption from the difficulties and trials inseparable from a dependence on medical practice; attaining to an enviable position as a practitioner and teacher, and devoting himself assiduously to the duties incident thereto, he has accomplished, in addition, an amount of scientific and literary labor, which may serve to exemplify how much can be effected by a determined purpose and unremitting industry, in spite of the discouragements and drawbacks arising from the distracting, irregular and almost incessant claims which the busy practitioner often pleads as a sufficient excuse for undertaking nothing beyond the practical exercise of his avocation.

We might point to the example of Prof. Gross, as in another aspect worthy of imitation for those aspiring to usefulness in a career of authorship. Not content with having accomplished enough to satisfy an honorable desire for distinction, he does not tire in laboring for the interests of his profession. At the present moment, not resting after the completion of the treatise which we have inadequately noticed in this brief article, he is busily engaged in the composition of a great work on surgery, which will be the capital of a column more enduring than marble, and which will hereafter inspire many an earnest spirit in the ranks of the medical profession with a desire to follow in his footsteps.

For sale by PHINNEY & Co. *

